# In Silico Conformational Analysis of the Short-Sequence Hypomurocin A Peptides

**DOI:** 10.1155/2015/281065

**Published:** 2015-01-28

**Authors:** Zoltán Násztor, János Horváth, Balázs Leitgeb

**Affiliations:** ^1^Institute of Biophysics, Biological Research Centre, Hungarian Academy of Sciences, Temesvári Körút 62, Szeged 6726, Hungary; ^2^Department of Medical Chemistry, Faculty of Medicine, University of Szeged, Dóm Tér 8, Szeged 6720, Hungary; ^3^Department of Microbiology, Faculty of Science and Informatics, University of Szeged, Közép Fasor 52, Szeged 6726, Hungary

## Abstract

In this theoretical study, a conformational analysis was performed on short-sequence hypomurocin A peptides, in order to identify their characteristic structural properties. For each hypomurocin A molecule, not only the backbone conformations, but also the side-chain conformations were examined. The results indicated that certain tetrapeptide units could be characterized by types I and III *β*-turn structures, and considering the helical conformations, it could be concluded that the hypomurocin A peptides showed a preference for the 3_10_-helical structure over the *α*-helical structure. Beside the backbone conformations, the side-chain conformations were investigated, and the preferred rotamer states of the side-chains of amino acids were determined. Furthermore, the occurrence of *i* ← *i* + 3 and *i* ← *i* + 4 intramolecular H-bonds was studied, which could play a role in the structural stabilization of *β*-turns and helical conformations. On the whole, our theoretical study supplied a comprehensive characterization of the three-dimensional structure of short-sequence hypomurocin A peptides.

## 1. Introduction

Hypomurocin (HM) peptides are the members of Peptaibol family [1–3], which were originally isolated from the fungus* Hypocrea muroiana* (synonymous to* Trichoderma atroviride*) [4], and they showed antibiotic and hemolytic activities. In a recent study, the HM molecules were also detected in other* Hypocrea spp.* [5], and based on the results, it was suggested that the production of peptaibols by* Trichoderma/Hypocrea* species may contribute to the colonization and defense of ecological niches for these fungi. Based on their sequence length, the hypomurocins can be divided into two distinct groups, namely, the short-sequence HM A peptides composed of 11 amino acids and the long-sequence HM B peptides consisting of 18 amino acid residues [4]. The former group of HM molecules covers six different peptides (see Table 1), which contain a nonproteinogenic amino acid (i.e., *α*-aminoisobutyric acid, Aib), and additionally, one of them (i.e., HM A-2 molecule) contains a further nonproteinogenic residue (i.e., D-isovaline, D-Iva). As it can be seen, the N-terminal amino acid of HM A peptides is acetylated, and an amino alcohol (i.e., leucinol, Leuol) is linked by an amide bond at their C-terminal end. Among the HM A molecules, for the HM A-1 peptide, the NMR measurement performed in dimethyl sulfoxide (DMSO) revealed that this molecule adopted a mixed helical conformation, which contained *α*- and 3_10_-helices, as well as type I *β*-turn structure [6]. Nevertheless, this NMR measurement led to the observation that the HM A-1 peptide could be characterized predominantly by 3_10_-helical structure in the presence of micelles [6]. In a subsequent NMR study, the structural features of HM A-3 and HM A-5 molecules were investigated in DMSO solution, and based on the results, it was suggested that these peptides possessed also a mixed helical conformation containing not only *α*- and 3_10_-helical parts, but also type I *β*-turn structure [7]. In our theoretical study, a detailed conformational analysis was performed on all six HM A peptides, in order to identify their characteristic structural properties, focusing on the backbone and side-chain conformations, as well as on the evolving intramolecular H-bonds.

## 2. Methods

The HM A molecules contain nonproteinogenic amino acids, such as Aib and D-Iva residues, and furthermore, they contain an amino alcohol, that is, Leuol; thus these nonstandard residues were parameterized by means of quantum chemical calculations. For the parameterization, the second-order Møller-Plesset perturbation method and the 6-311G basis set were applied. In order to derive the partial atomic charges for the above-mentioned nonstandard residues, the restrained electrostatic potential (RESP) method was used.

To perform the conformational analysis of HM A peptides, the simulated annealing (SA) method was applied, as it was previously used for the short-sequence trichobrachin molecules [8]. The SA calculations were carried out with the AMBER 9 program [9], in the course of which the AMBER 99SB force field [10] and the Generalized Born implicit solvent model [11–13] were applied, and no-cutoff was used for the nonbonding interactions. In the case of each HM A molecule, first of all an initial energy-minimization was performed, and using this geometrically optimized structure, the SA procedure composed of three consecutive stages was applied, as follows: (1) heating to 1000 K for 1000 fs, (2) equilibration at 1000 K during 4000 fs, and (3) cooling from 1000 K to 50 K for 10000 fs. For the cooling stage, a near-exponential protocol was used, which consisted of the following linear phases, successively: (1) from 1000 K to 500 K during 1000 fs, (2) from 500 K to 200 K for 2000 fs, (3) from 200 K to 50 K during 7000 fs. The SA procedure, composed of heating, equilibration, and cooling stages, was carried out 1000 times; thus 1000 conformers were obtained in the case of each peptide, for which a final energy-minimization was performed. This geometry optimization was carried out by the steepest descent method applied for the first 100 steps, which was followed by the conjugated gradient method, using the gradient convergence criterion of 0.001 kcal mol^−1^ Å^−1^ and the maximum number of iterations of 10000.

## 3. Results and Discussion

In order to identify the characteristic structural features of HM A peptides, the backbone and side-chain conformations were studied, and the evolving intramolecular H-bonds were investigated, based on the conformers derived from the SA calculations.

To characterize the backbone conformations of HM A molecules, the presence of types I and III *β*-turns was examined along the entire sequence of peptides. For the determination of *β*-turn structures, the typical ranges of Φ and Ψ torsion angles with regard to the *i* + 1th and *i* + 2th residues of a tetrapeptide unit were used [14, 15]. They were as follows: (1) in the case of type I *β*-turn: Φ_*i*+1_ = −60° ± 30° and Ψ_*i*+1_ = −30° ± 30°, Φ_*i*+2_ = −90° ± 30° and Ψ_*i*+2_ = 0° ± 30°; (2) in the case of type III *β*-turn: Φ_*i*+1_ = −60° ± 30° and Ψ_*i*+1_ = −30° ± 30°, Φ_*i*+2_ = −60° ± 30° and Ψ_*i*+2_ = −30° ± 30°. The populations of types I and III *β*-turns identified in certain tetrapeptide units of HM A molecules are represented in Table 2 for the conformers obtained by the SA simulations. Based on these results, it could be concluded that types I and III *β*-turns could be detected for almost all tetrapeptide units of each molecule. In the case of five HM A peptides (i.e., HM A-1, HM A-3, HM A-4, HM A-5, and HM A-5a), a similar conformational pattern could be observed with regard to the populations of *β*-turn structures. Accordingly, for the Gln^2^–Xaa^3^–Xaa^4^–Aib^5^ and Pro^6^–Leu^7^–Xaa^8^–Aib^9^ tetrapeptide segments, larger amount of types I and III *β*-turns could be found, in comparison with those detected in the case of other four tetrapeptide units (i.e., Xaa^1^–Gln^2^–Xaa^3^–Xaa^4^, Xaa^4^–Aib^5^–Pro^6^–Leu^7^, Aib^5^–Pro^6^–Leu^7^–Xaa^8^, and Xaa^8^–Aib^9^–Pro^10^–Leuol^11^). On the basis of data shown in Table 2, it could be seen that, for the HM A-2 molecule, the relationship between the populations of *β*-turn structures was the opposite, considering the above-mentioned two groups of tetrapeptide segments. Consequently, types I and III *β*-turns appeared with lower frequency in the case of former two tetrapeptide units than for the latter four tetrapeptide segments. Nevertheless, taking into account the different tetrapeptide units, it could be seen that, in the case of HM A-2 molecule, the amount of *β*-turn structures found in the Gln^2^–Xaa^3^–Xaa^4^–Aib^5^ and Pro^6^–Leu^7^–Xaa^8^–Aib^9^ segments were lower, while the populations of types I and III *β*-turns observed in the Xaa^4^–Aib^5^–Pro^6^–Leu^7^, Aib^5^–Pro^6^–Leu^7^–Xaa^8^ and Xaa^8^–Aib^9^–Pro^10^–Leuol^11^ units were larger, as compared to those detected in the corresponding tetrapeptide segments of other five HM A molecules. On the whole, these results indicated that the HM A peptides could be characterized by types I and III *β*-turn structures.

**Figure 1:**
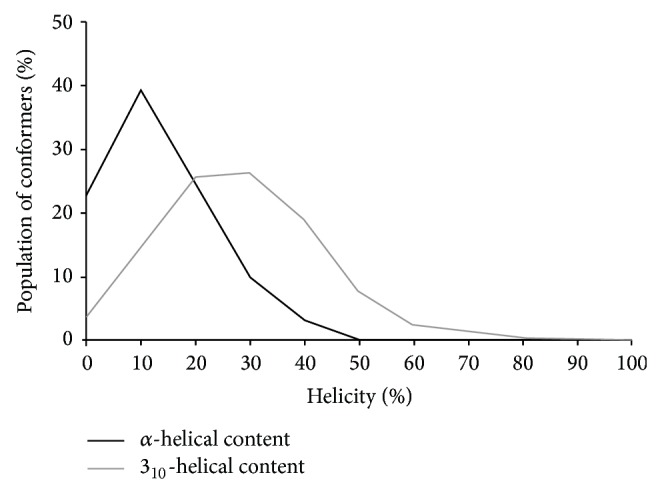
Distributions of the helicity values for the HM A-1 peptide.

In order to further describe the backbone conformations of HM A molecules, the occurrence of 3_10_- and *α*-helical conformations was also investigated. To identify these helical structures, the following characteristic ranges of Φ and Ψ torsion angles were applied: (1) for the 3_10_-helix, Φ = −60° ± 30° and Ψ = −30° ± 30° [16]; (2) for the *α*-helix, Φ = −60° ± 30° and Ψ = −50° ± 30° [16]. In the case of two types of the helical conformations mentioned above, the typical ranges of Φ and Ψ torsion angles overlap with each other; thus the cumulative helical content was also calculated, using the combined ranges of Φ and Ψ torsion angles characteristic to the 3_10_- and *α*-helices. In order to characterize the evolving helical structures, the percentages of helical contents (i.e., helicities) were calculated for each conformer derived from the SA calculations, applying the following formula [17, 18]:

(1) $$\begin{array}{c} f=\frac{n_{h}}{N}\cdot 100, \end{array}$$

where *f* is the helicity and *n*_*h*_ is the number of amino acids satisfying the torsion angle criteria for the 3_10_- and *α*-helical conformations and the cumulative helical content, respectively. For simplicity, in this study, *N* is not equal to the number of all amino acids (i.e., 11 residues) found in the sequences of HM A peptides, as it was previously used [17, 18]. In the present study, the *N* = 10 was applied, and the reasons for this were as follows: (1) the short sequence length of HM A molecules; (2) the Ψ torsion angle could not be defined in the case of Leuol^11^ residue. Based on the conformers obtained by the SA simulations, the distributions of helicity values were calculated, and the populations of conformers characterized by certain helical contents were determined. As a representative sample, the distributions of helicity values are illustrated in Figure 1 for the HM A-1 peptide, and these distributions were found to be similar in the case of other five molecules. Nevertheless, the populations of conformers possessing different helical contents are shown in Table 3 for each HM A peptide. Since the values regarding the cumulative helical content proved to be equal to the values concerning the 3_10_-helical content, the distribution of these helicity values was not demonstrated in Figure 1, and these helicity values were not included in Table 3. As the distribution plot and the values with regard to the 3_10_- and *α*-helical contents indicated, differences could be observed between these two types of helicities. Accordingly, larger populations of conformers characterized by at least 30% helicity could be detected for the 3_10_-helical content, as compared to those found in the case of *α*-helical content. Moreover, on the basis of data presented in Table 3, it could be concluded that a similar distribution of helicity values could be observed for all the HM A molecules, taking into account the 3_10_- and *α*-helical contents, respectively. On the basis of these results, it could be suggested that the HM A peptides showed a preference for the 3_10_-helical structure over the *α*-helical conformation.

For the side-chains of amino acids, except for the Aib and Pro residues, the proportions of three rotamer states (i.e., *g*(+), *g*(−), and* trans*) were determined (see Table 4), and the preferred rotamers were identified, on the basis of conformers derived from the SA calculations. The side-chain of the D-Iva^1^ amino acid of HM A-2 peptide showed a preference for the *g*(+) rotamer over the other two rotamer states; nevertheless, the ratios of *g*(−) and* trans* rotamers proved to be similar. In contrast, for the side-chains of Gln^2^, Val^3^, and Val^4^ residues, mainly the *g*(−) rotamer state was favored, and similar proportions of *g*(+) and* trans* rotamers could be observed in the majority of cases. For the side-chains of Ile^3^, Ile^4^, and Ile^8^ amino acids, a decreasing tendency could be detected with respect to the ratios of three rotamer states, as follows: *g*(+) >* trans* > *g*(−). In the case of the side-chains of Leu^4^, Leu^7^, and Leu^8^ residues, similar proportions of *g*(−) and* trans* rotamers could be observed in the majority of cases, and both of them were found to be preferred over the *g*(+) rotamer state. For the side-chains of Leuol^11^ amino acids, large, moderate, and small ratios were identified concerning the *g*(−),* trans,* and *g*(+) rotamers, respectively.

Since various intramolecular H-bonds could contribute to the stability of the conformational states of peptides, the appearance of H-bonds evolved between the backbone NH donor and CO acceptor groups was investigated. Among them, the *i* ← *i* + 3 H-bonds formed between a NH group of *i* + 3th and a CO group of *i*th amino acids play an important role in the structural stabilization of *β*-turns, as well as of 3_10_-helical conformation. The other type of intramolecular H-bonds, such as the *i* ← *i* + 4 H-bonds formed between a NH group of *i* + 4th and a CO group of *i*th residues, contributes to the structural stability of *α*-helical conformation. The occurrence of *i* ← *i* + 3 and *i* ← *i* + 4 H-bonds was examined along the entire sequence of HM A peptides. An intramolecular H-bond was assumed to exist if the *N* ⋯ *O* distance between the N atom of NH donor group and the O atom of CO acceptor group was within 3.5 Å, and if the *N*–*H* ⋯ *O* angle subtended at the H atom by the bond to the N atom and the line joining the H and O atoms was larger than 120°. The populations of the different types of *i* ← *i* + 3 and *i* ← *i* + 4 H-bonds observed for the conformers of each HM A molecule are represented in Table 5. These results indicated that the *i* ← *i* + 3 H-bonds appeared in all the tetrapeptide units, for which types I and III *β*-turns were identified. The *i* ← *i* + 4 H-bonds were also detected along the entire sequence of molecules; however, their populations proved to be much smaller, as compared to those in the case of *i* ← *i* + 3 H-bonds. Based on these results, it could be also suggested that the HM A peptides could be characterized by *β*-turn or 3_10_-helical structure rather than by *α*-helical conformation.

## 4. Conclusions

In the present study, a conformational analysis was performed for all the HM A peptides, and the structural properties of these short-sequence peptaibols were characterized comprehensively. Taking into account the backbone conformations, types I and III *β*-turns were identified in certain tetrapeptide units of each HM A molecule, and additionally, the 3_10_- and *α*-helical contents were determined. These results indicated that the HM A peptides could be characterized by *β*-turn structures or 3_10_-helical conformation rather than by *α*-helical structure. Nevertheless, the side-chain conformations of amino acids were investigated, and the ratios of three rotamer states, as well as the preferred rotamers, were identified. For the HM A peptides, the appearance of *i* ← *i* + 3 and *i* ← *i* + 4 H-bonds was examined, and the results led to the conclusion that the *i* ← *i* + 3 H-bonds could contribute to the stability of *β*-turn and 3_10_-helical structures. As it was mentioned previously, based on the data derived from the NMR measurements, it was suggested that the HM A-1, HM A-3, and HM A-5 peptides adopted a mixed helical conformation, for which *α*- and 3_10_-helices, as well as type I *β*-turn structure, appeared [6, 7]. The results obtained by our structural investigation indicated that the HM A molecules could be characterized by types I and III *β*-turns or 3_10_-helical conformation, and these peptides showed a preference for these two secondary structural elements over the *α*-helical structure. In summary, our theoretical study provided a detailed description of the three-dimensional structure of short-sequence HM A peptides.

## Figures and Tables

**Table 1:** Sequences of the HM A peptides.

Peptides	Sequences of peptides
HM A-1	Ac–Aib^1^–Gln^2^–Val^3^–Val^4^–Aib^5^–Pro^6^–Leu^7^–Leu^8^–Aib^9^–Pro^10^–Leuol^11^
HM A-2	Ac–D-Iva^1^–Gln^2^–Val^3^–Val^4^–Aib^5^–Pro^6^–Leu^7^–Leu^8^–Aib^9^–Pro^10^–Leuol^11^
HM A-3	Ac–Aib^1^–Gln^2^–Val^3^–Leu^4^–Aib^5^–Pro^6^–Leu^7^–Ile^8^–Aib^9^–Pro^10^–Leuol^11^
HM A-4	Ac–Aib^1^–Gln^2^–Ile^3^–Val^4^–Aib^5^–Pro^6^–Leu^7^–Leu^8^–Aib^9^–Pro^10^–Leuol^11^
HM A-5	Ac–Aib^1^–Gln^2^–Ile^3^–Ile^4^–Aib^5^–Pro^6^–Leu^7^–Leu^8^–Aib^9^–Pro^10^–Leuol^11^
HM A-5a	Ac–Aib^1^–Gln^2^–Ile^3^–Leu^4^–Aib^5^–Pro^6^–Leu^7^–Ile^8^–Aib^9^–Pro^10^–Leuol^11^

**Table 2:** Populations (in %) of the types I and III *β*-turns identified in certain tetrapeptide units of the HM A molecules. Xaa^1^ = Aib/D-Iva, Xaa^3^ = Val/Ile, Xaa^4^ = Val/Leu/Ile, and Xaa^8^ = Leu/Ile.

Types I and III *β*-turns						
Tetrapeptide units	HM A-1	HM A-2	HM A-3	HM A-4	HM A-5	HM A-5a
Xaa^1^–Gln^2^–Xaa^3^–Xaa^4^	9.6	8.9	8.6	8.6	8.3	11.5
Gln^2^–Xaa^3^–Xaa^4^–Aib^5^	17.1	5.2	17.7	15.7	16.2	15.1
Xaa^4^–Aib^5^–Pro^6^–Leu^7^	6.1	9.6	6.3	4.4	5.7	5.8
Aib^5^–Pro^6^–Leu^7^–Xaa^8^	7.1	11.3	9.6	7.2	7.9	8.7
Pro^6^–Leu^7^–Xaa^8^–Aib^9^	14.5	7.4	17.1	13.4	11.6	12.4
Xaa^8^–Aib^9^–Pro^10^–Leuol^11^	8.6	12.9	8.2	8.9	7.6	9.1

**Table 3:** Populations (in %) of the conformers characterized by the different helical contents for the HM A peptides.

Helicity	HM A-1	HM A-2	HM A-3	HM A-4	HM A-5	HM A-5a
3_10_-helical content						
0%	3.6	3.7	3.8	3.1	4.1	2.5
10%	14.4	15.2	15.2	14.9	15.5	14.8
20%	25.8	23.2	26.3	28.2	24.9	24.5
30%	26.2	24.0	25.4	24.6	28.3	26.0
40%	18.7	18.8	17.5	17.8	16.2	19.0
50%	7.6	10.2	7.4	8.7	7.7	9.6
60%	2.3	3.7	3.5	2.4	2.6	2.7
70%	1.2	1.1	0.7	0.2	0.6	0.9
80%	0.2	0.1	0.2	0.1	0.1	0.0

*α*-helical content						
0%	22.5	14.9	23.8	23.0	21.0	19.2
10%	39.4	33.6	35.6	37.5	39.7	37.5
20%	24.5	27.7	29.1	27.3	27.3	27.4
30%	10.1	17.2	9.5	9.7	8.8	12.3
40%	3.1	4.6	1.4	2.1	2.8	2.7
50%	0.3	1.7	0.5	0.3	0.4	0.8
60%	0.1	0.3	0.1	0.1	0.0	0.1

**Table 4:** Proportions (in %) of the g(+), g(−), and *trans* rotamers for the side-chains of the amino acids of HM A peptides. Xaa^3^ = Val/Ile, Xaa^4^ = Val/Leu/Ile, and Xaa^8^ = Leu/Ile.

Rotamers	D-Iva^1^	Gln^2^	Xaa^3^	Xaa^4^	Leu^7^	Xaa^8^	Leuol^11^
HM A-1							
g(+)	—	22.0	22.0	20.1	14.4	14.5	16.3
g(−)	—	54.5	50.3	51.3	48.5	47.5	51.1
*Trans *	—	23.5	27.7	28.6	37.1	38.0	32.6

HM A-2							
g(+)	45.7	19.7	22.2	20.9	15.1	14.7	13.1
g(−)	30.0	52.2	51.5	52.6	46.5	48.0	57.5
*Trans *	24.3	28.1	26.3	26.5	38.4	37.3	29.4

HM A-3							
g(+)	—	18.8	21.7	13.3	14.7	42.2	15.9
g(−)	—	57.8	48.9	46.6	46.1	23.6	50.5
*Trans *	—	23.4	29.4	40.1	39.2	34.2	33.6

HM A-4							
g(+)	—	20.1	49.0	24.4	17.0	15.5	14.4
g(−)	—	52.7	21.4	46.5	44.5	45.2	53.6
*Trans *	—	27.2	29.6	29.1	38.5	39.3	32.0

HM A-5							
g(+)	—	18.6	47.6	41.5	17.3	15.5	14.8
g(−)	—	55.3	22.4	24.0	46.3	45.4	50.3
*Trans *	—	26.1	30.0	34.5	36.4	39.1	34.9

HM A-5a							
g(+)	—	21.9	40.5	15.6	15.9	43.4	13.8
g(−)	—	52.7	23.7	43.4	43.5	19.5	53.5
*Trans *	—	25.4	35.8	41.0	40.6	37.1	32.7

**Table 5:** Populations (in %) of the different types of *i* ← *i* + 3 and *i* ← *i* + 4 H-bonds for the HM A peptides. Xaa^1^ = Aib/D-Iva, Xaa^4^ = Val/Leu/Ile, and Xaa^8^ = Leu/Ile.

Intramolecular H-bonds						
*i* ← *i* + 3 H-bonds	HM A-1	HM A-2	HM A-3	HM A-4	HM A-5	HM A-5a

Xaa^1^← Xaa^4^	11.4	13.1	14.0	12.7	10.8	13.3
Gln^2^← Aib^5^	10.0	8.8	12.9	10.0	9.3	10.1
Xaa^4^← Leu^7^	8.0	12.3	9.3	6.2	7.9	7.2
Aib^5^← Xaa^8^	12.2	16.2	11.2	13.2	11.5	9.2
Pro^6^← Aib^9^	11.9	11.2	11.2	10.0	11.1	10.4
Xaa^8^← Leuol^11^	8.7	16.7	7.7	7.3	7.5	8.7

*i* ← *i* + 4 H-bonds	HM A-1	HM A-2	HM A-3	HM A-4	HM A-5	HM A-5a

Xaa^1^← Aib^5^	2.9	3.2	2.3	2.5	1.5	2.9
Xaa^3^← Leu^7^	1.1	0.1	1.1	1.5	0.7	1.6
Xaa^4^← Xaa^8^	1.2	2.7	1.2	1.3	1.9	1.2
Aib^5^← Aib^9^	2.5	5.6	3.1	2.8	3.2	1.5
Leu^7^← Leuol^11^	0.9	0.6	1.4	1.3	1.0	1.9
